# Tumor-Induced Oblique Abdominal Muscle Paralysis Syndrome: A Case of Position-Dependent Abdominal Distension and Umbilical Displacement

**DOI:** 10.7759/cureus.75341

**Published:** 2024-12-08

**Authors:** Yojiro Ishikawa, Keishi Sakai, Satoshi Teramura, Kengo Ito, Takayuki Yamada

**Affiliations:** 1 Radiology, Tohoku Medical and Pharmaceutical University, Sendai, JPN; 2 College of Medicine, Tohoku Medical and Pharmaceutical University, Sendai, JPN

**Keywords:** abdominal muscle paralysis, displacement of the umbilicus, malignant pleural mesothelioma, tumor invasion-induced abdominal muscle paralysis syndrome, unilateral paralysis of the rectus abdominis muscle

## Abstract

Unilateral paralysis of the oblique abdominal muscles is commonly linked to non-malignant conditions, such as herniated discs, shingles, and phrenic nerve injury, each leading to localized muscle weakness through nerve impairment. However, oblique abdominal muscle paralysis caused by malignant tumors is exceedingly rare. This report presents a unique case of left oblique abdominal muscle paralysis induced by malignant pleural mesothelioma (MPM), demonstrating a distinct pattern of position-dependent abdominal bulging and umbilical displacement. The patient, a male in his 70s, was initially treated with chemotherapy for left-sided MPM and later received radiation therapy (RT) for pleural lesions causing pain. As the disease progressed, he developed a visible bulging of the left lateral abdomen, which was more pronounced in upright positions (standing or sitting) and resolved in the supine position. CT imaging showed atrophy of the left oblique abdominal muscle and a displacement of the umbilicus to the right, findings were consistent with intercostal nerve impairment due to tumor invasion. This case underscores the importance of conducting abdominal examinations in various positions when nerve impairment by a tumor is suspected. The presence of position-dependent lateral abdominal bulging, lateral displacement of the umbilicus, and lack of intraperitoneal abnormalities should raise suspicion for tumor-induced oblique abdominal muscle paralysis. We propose defining such cases as “tumor-induced oblique abdominal muscle paralysis syndrome” to improve diagnostic accuracy and aid in the effective management of malignancy-associated abdominal neuropathies.

## Introduction

Unilateral paralysis of the abdominal muscles (including the oblique abdominal muscles) is primarily caused by non-malignant factors, such as herniated discs [1], shingles [2], and phrenic nerve injury [3]. A herniated disc, particularly in the lumbar or thoracic spine, can lead to muscle weakness and motor impairment in areas controlled by the affected nerve. For instance, a lumbar herniation compressing the L2-L3 nerve roots may result in unilateral paralysis of the abdominal muscles, including the oblique muscles [1]. Similarly, abdominal muscle paralysis involving the oblique muscles associated with shingles occurs when nerve function is impaired due to viral reactivation within the spinal ganglia [2]. Even without typical skin symptoms or pain, unilateral abdominal muscle paralysis may occur temporarily, highlighting an important clinical consideration. Additionally, phrenic nerve damage from surgery or trauma has been reported to cause unilateral diaphragmatic paralysis, which can extend to the abdominal muscles, particularly the oblique muscles [3].

In contrast, mechanisms by which malignant tumors cause abdominal muscle paralysis, specifically involving the oblique muscles, remain sparsely documented, and a comprehensive understanding of this condition is lacking. While malignant tumors frequently affect the nervous system through local invasion or distant metastasis [4,5], resulting in nerve paralysis and sensory disturbances, reports of abdominal muscle paralysis, particularly involving the oblique muscles, due to malignancy are exceedingly rare. There is limited literature on how a malignant tumor may directly invade nerves controlling the oblique abdominal muscles, thereby causing unilateral paralysis.

This case represents a rare clinical example of malignant pleural mesothelioma (MPM) involvement leading to unilateral abdominal muscle paralysis, with paralysis explicitly localized to the oblique muscles in the abdominal region. Here, we describe in detail the clinical significance and diagnostic considerations for this unique case of tumor-induced unilateral oblique abdominal muscle paralysis.

## Case presentation

**Figure 1:**
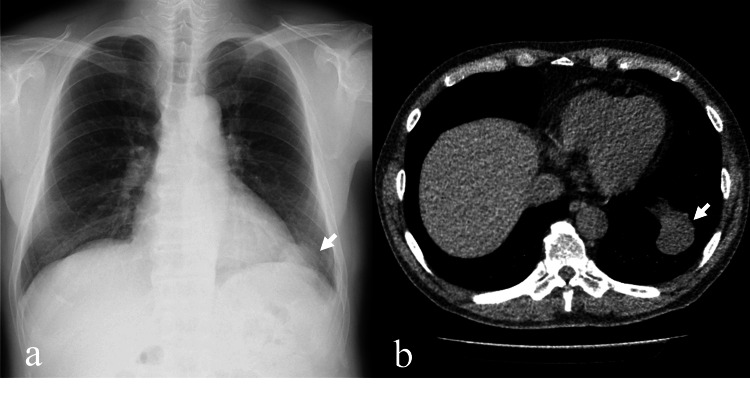
Chest X-ray and CT images at initial diagnosis (a) The chest X-ray revealed a nodular shadow in the left lower lung field (arrow). (b) The chest CT showed a 3.7 cm mass with clear boundaries in the left lower lobe, directly adjacent to the diaphragm (arrow).

A male patient in his 70s presented with a nodular shadow in the left lung field on chest radiography. Subsequent CT scan revealed a 3.7 cm mass in the left lower lobe adjacent to the diaphragm (Figures 1a-1b) and a 1.7 cm mass in the lingula, both closely associated with the pleura. These findings raised the suspicion of pleural invasion and possible pleural spread. A thoracoscopic lung biopsy was then performed, confirming the diagnosis of MPM.

Based on the imaging and histopathological findings, the disease was staged as stage IV due to the multifocal pleural involvement and the risk of intrathoracic spread, despite the absence of distant metastases. Given the advanced stage and pleural spread, surgical intervention was not considered feasible, leading to a treatment plan centered on chemotherapy.

He initially received four cycles of cisplatin (CDDP; 75 mg/m²) and pemetrexed (PEM; 500 mg/m²), followed by PEM monotherapy (500 mg/m²) administered every three weeks. He completed 25 courses of PEM, commencing one year after diagnosis and continuing until the third year. In the third year following diagnosis, the patient commenced nivolumab therapy (200 mg every two weeks), which was continued until the fifth year (50 courses).

**Figure 2:**
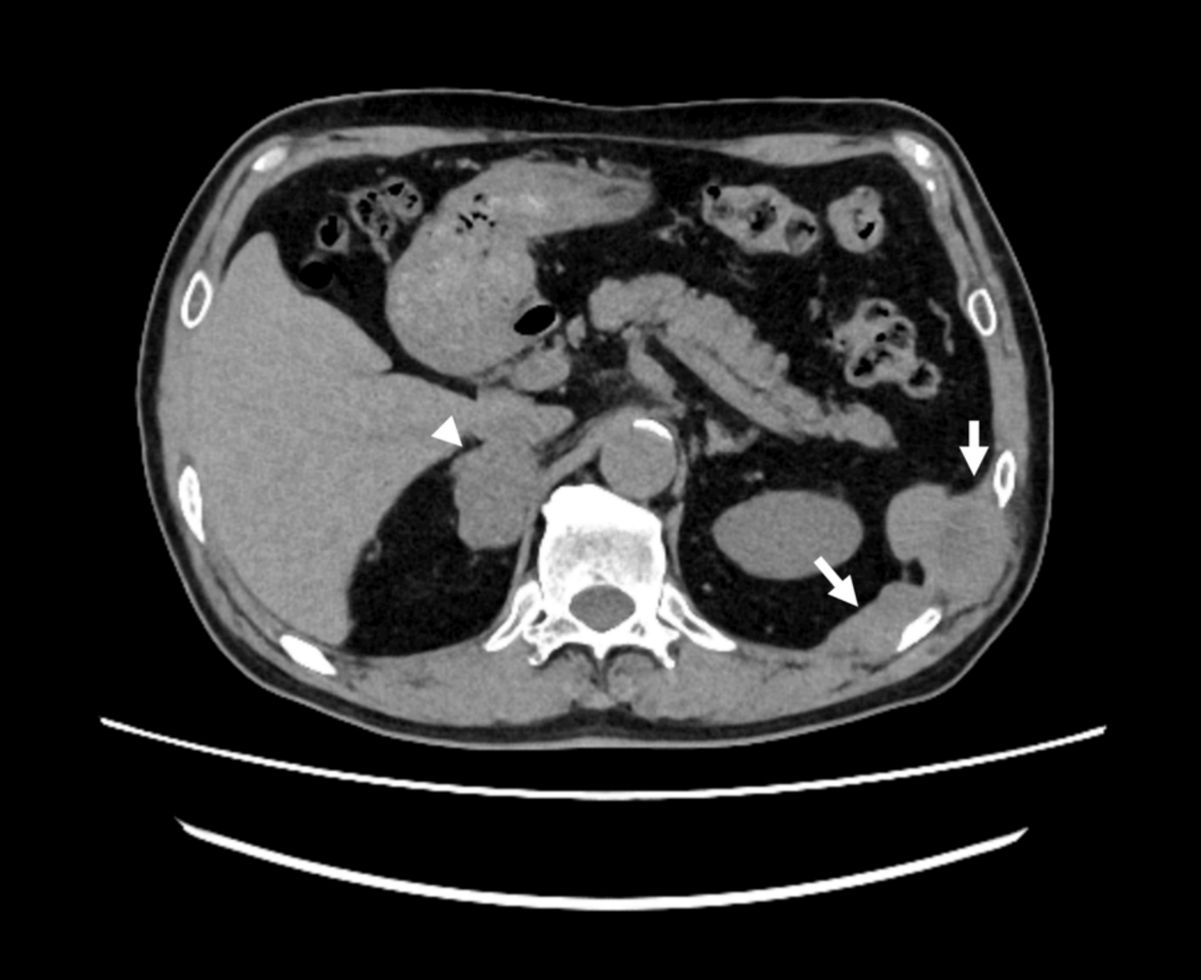
Abdominal CT image after chemotherapy The multiple pleural lesions exhibited increased size and extended specifically between the 10th and 11th intercostal spaces, resulting in abdominal discomfort on the left side (arrows). Additionally, metastasis to the right adrenal gland became evident on imaging (arrowhead), indicating further progression of the disease.

Chemotherapy was administered, but the pleural lesions gradually increased in size and a right adrenal metastasis appeared. The patient's primary complaint was abdominal discomfort in the left abdominal region. A CT scan revealed the presence of a lesion in the pleura in the vicinity of the 10th and 11th ribs (Figure 2).

**Figure 3:**
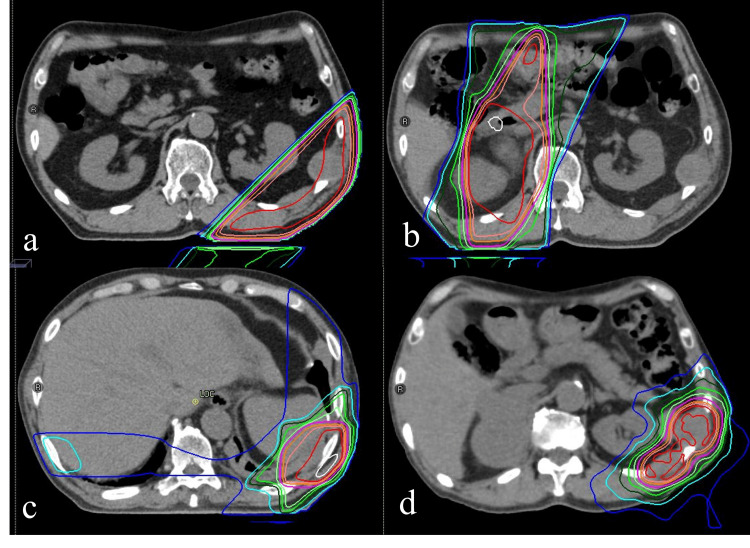
Radiation planning and dose distribution All treatment plans were created using RayStation (RaySearch Laboratories, Stockholm, Sweden). (a) A total dose of 39 Gy in 13 fractions was delivered to the pleural lesions between the 10th and 11th intercostal spaces on the left chest wall. (b) Four months later, a total dose of 30 Gy in 10 fractions was administered to the metastatic lesion in the right adrenal gland. (c) During the same period, a dose of 20 Gy in five fractions was delivered to the pleural lesion in the ninth intercostal space. (d) Approximately six to nine months after the initial treatment, intensity-modulated radiation therapy (IMRT) was performed to re-irradiate the pleural lesions in the 10th and 11th intercostal spaces, delivering an additional dose of 25 Gy in 10 fractions to address the recurrence of pain.

Radiation therapy (RT) was initially administered to pleural lesions in the 10th and 11th intercostal spaces on the left chest wall using three-dimensional conformal radiation therapy (3DCRT), delivering a total dose of 39 Gy in 13 fractions, which effectively alleviated the pain. Four months later, 3DCRT was performed to treat the right adrenal gland, delivering 30 Gy in 10 fractions. During the same period, 20 Gy in five fractions was administered to pleural lesions in the ninth intercostal space (Figures 3a-3c), where pain had recurred. Five months after these treatments, corresponding to nine months from the initial RT, intensity-modulated radiation therapy (IMRT) was used to re-irradiate the pleural lesions in the 10th and 11th intercostal spaces, delivering a total dose of 25 Gy in 10 fractions to address the recurrence of pain in this area (Figure 3d).

**Figure 4:**
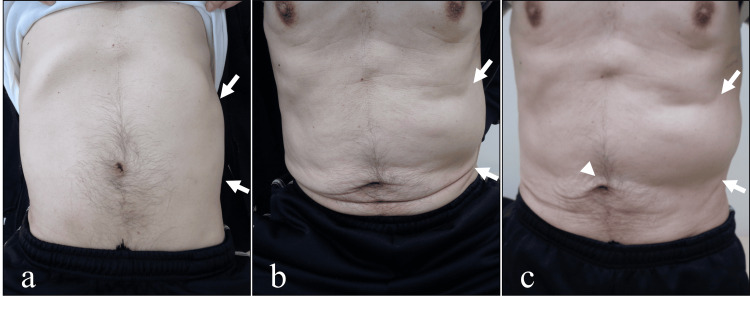
Macroscopic findings of the abdomen (a) Supine position findings: No visible abdominal distention or palpable mass was detected (arrows). The abdomen was flat and soft, without tenderness or localized areas of concern. (b) Sitting position findings: A notable bulge was observed on the left side of the abdomen, associated with localized discomfort in the same region (arrows). (c) Standing position findings: The bulge on the left side of the abdomen was more pronounced than in the supine or sitting positions (arrows), and abdominal discomfort was exacerbated. Additionally, the umbilicus was displaced toward the unaffected side relative to the supine and sitting positions (arrowhead).

Around six to nine months after the initial RT, the patient developed a bulging of the left abdomen. Despite the patient’s abdominal complaints, CT imaging did not reveal any clear abnormalities, and the abdomen appeared flat in the recumbent position (Figure 4a), with the bulging notably present only when standing or sitting. Upon standing, a shift of the navel to the right was observed (Figures 4b-4c).

**Figure 5:**
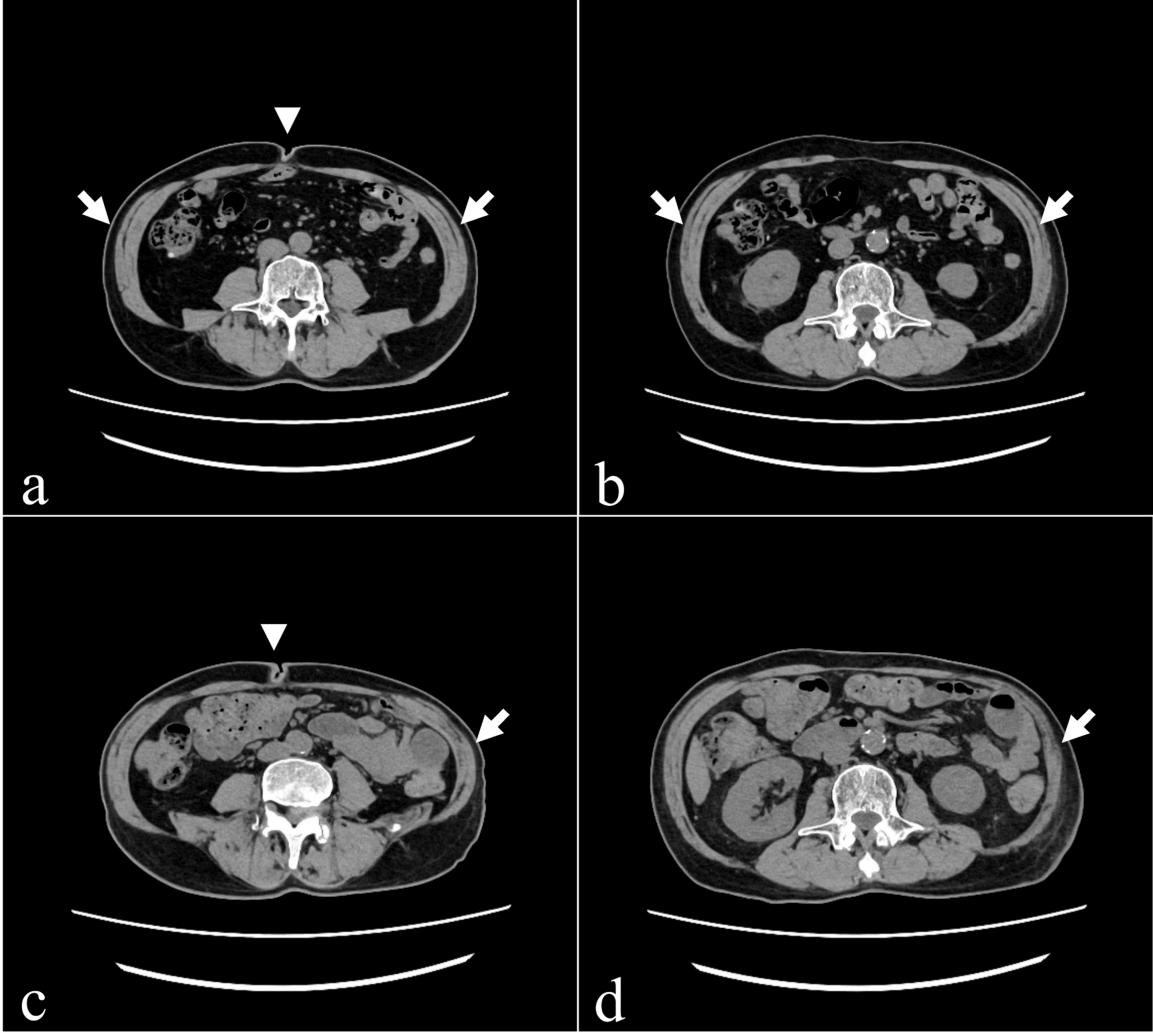
Comparison of abdominal CT images before and after irradiation (a) The initial abdominal CT image obtained at the start of radiation therapy reveals no apparent asymmetry between the left and right oblique muscles at the level of the umbilicus (arrows), with the umbilicus positioned near the midline but slightly shifted to the right (arrowhead). (b) At the level of the lower pole of the kidney, a similar observation is noted, with no discernible difference in the oblique abdominal muscles bilaterally (arrows). (c) In contrast, the abdominal CT image taken post-irradiation demonstrates thinning of the left oblique abdominal muscle at the level of the umbilicus (arrow). Additionally, a slight displacement of the umbilical region to the right is observed, indicating a shift toward the unaffected side (arrowhead). (d) At the level of the lower pole of the kidney, further evidence of thinning in the left oblique abdominal muscle is also noted (arrow).

While the CT scan did not reveal any significant intra-abdominal abnormalities, atrophy of the left external oblique abdominal muscle and left internal oblique abdominal muscle was observed, along with a rightward displacement of the navel. These findings were attributed to paralysis of the left oblique abdominal muscles due to impairment of the intercostal nerve (Figures 5a-5d), which caused abdominal bulging when the patient was upright. The diagnosis was based on the absence of other definitive findings indicative of alternative conditions such as shingles or diabetic neuropathy. No vesicular rash, which is characteristic of shingles, was observed during the clinical course, and the patient had no history of diabetes or symptoms suggesting diabetic neuropathy. Additionally, imaging studies revealed no abnormalities aside from the tumor, and there were no findings of infectious or metabolic processes that could explain the symptoms. Symptomatic relief was partially achieved by regulating bowel movements and reducing intestinal gas; however, the symptoms persisted post-irradiation.

## Discussion

This is a rare case of intercostal nerve paralysis caused by MPM, leading to unilateral paralysis of the oblique abdominal muscles, abdominal distension, and displacement of the umbilicus toward the unaffected side. MPM is a tumor that develops on the serous surface of the pleura, peritoneum, or pericardium and is primarily caused by asbestos exposure. Patients often suffer from pain and respiratory distress due to direct tumor invasion of the chest wall, lungs, vertebrae, and intercostal nerves [6]. Among patients with MPM, rib metastasis (destruction) is relatively rare, with a reported incidence of approximately 12% [7]. The umbilical displacement resembles the phenomenon observed in unilateral facial paralysis, where the corner of the mouth shifts toward the unaffected side [8]. The literature supports that abdominal muscle paralysis can impact the position of the umbilicus. The beaver sign is known as the phenomenon of the navel moving upward when the trunk is flexed from the supine position and is used to indicate spinal cord lesions between T10 and T12 [9,10]. Moreover, it has been suggested that nerve damage due to acute abdominal disease or surgery may contribute to umbilical displacement and abdominal distension, indicating the critical role of abdominal wall muscles in trunk support [11].

In this case, despite the patient’s abdominal complaints, no clear abnormalities were found on imaging, and the abdomen appeared flat and supple during the supine examination, making symptom detection challenging. Without observing the patient seated or standing, the abdominal distension could have easily been overlooked, complicating symptom assessment and findings alignment. Typically, abdominal examinations are performed in the supine position [10,12]; however, in cases where tumor-induced nerve involvement results in position-dependent symptoms, examination in seated or standing positions may also be essential. For abdominal wall pseudo hernias associated with shingles, blisters, and scars appear along the affected nerve ganglia, simplifying the correlation with physical findings [13,14]. In contrast, with malignant tumors, the location of the tumor often does not match the abdominal symptoms, underscoring the necessity of integrating imaging, patient interviews, and thorough physical examinations [15].

When specific clinical features align, as in this case, tumor-induced oblique abdominal muscle paralysis should be considered. These features include (1) abdominal distension that intensifies in standing or sitting positions and decreases in the supine position, (2) lateral displacement of the umbilicus toward the unaffected side, (3) absence of detectable organic abnormalities on imaging within the intraperitoneal cavity, and (4) visible thinning of the oblique abdominal muscles. Defining cases with these findings as “tumor-induced oblique abdominal muscle paralysis syndrome” may improve recognition, diagnosis, and management. Explicitly identifying position-dependent symptoms such as abdominal distension and umbilical displacement caused by tumor-related neuropathy and training healthcare providers to recognize this syndrome could significantly enhance diagnostic precision and patient care [16,17]. Recently, there has been an increase in the number of papers on specific muscle weakness caused by malignant tumors [4,5,18,19]. However, it is possible that there is not yet sufficient accumulation of knowledge in this field. Malignant tumors have the potential to affect all muscles, including the oblique abdominal muscles, and their controlling nerves. Nevertheless, their clinical characteristics remain insufficiently clear.

While this case highlights the significant impact of tumor-induced nerve damage on the oblique abdominal muscles, it is important to acknowledge potential limitations. The patient received a relatively high radiation dose of 39 Gy in 13 fractions as part of the initial treatment. Although this dose is not considered excessively high for inducing late radiation toxicity, the possibility of radiation-induced nerve damage or muscle atrophy cannot be entirely excluded, as described in the literature [20]. However, given the timing of symptom onset (six to nine months after irradiation) and the close anatomical relationship between the tumor and the intercostal nerves, we believe that tumor-related effects, such as nerve infiltration or compression, likely played a more significant role in this case. As this is a single case report, definitive conclusions regarding the exact contributions of radiation versus tumor effects cannot be drawn. Further research and accumulation of similar cases are necessary to deepen the understanding of tumor-induced muscle paralysis and its differentiation from radiation-induced effects.

## Conclusions

This is a rare case of intercostal nerve palsy secondary to a malignant tumor, resulting in unilateral oblique abdominal muscle paralysis with displacement of the umbilicus to the healthy side and position-dependent abdominal distension. While standard abdominal examination is typically performed in the supine position, cases like this one underscore the necessity for a more multifaceted approach, including observation in seated or standing positions. If (1) abdominal distension intensifies in standing or seated positions and decreases in the supine position, (2) the umbilicus shifts toward the unaffected side, or (3) there is no organic abnormality on imaging with visible thinning of the oblique abdominal muscles, tumor-induced oblique abdominal muscle paralysis should be suspected. Defining such cases as “tumor-induced oblique abdominal muscle paralysis syndrome” would likely hold substantial diagnostic and therapeutic value in the management of malignant tumor-related abdominal wall neuropathy.
